# Supplementary material for: Ubiquitin-proteasome pathway annotation in Diaphorina citri can reveal potential targets for RNAi-based pest management
Source: GigaByte. 2022 Mar 2;2022:gigabyte43. doi: 10.46471/gigabyte.43 (PMC9933519; doi:10.46471/gigabyte.43)
Supplement: Peer review history

# Reviewer report: Review MS


Reviewer name and names of any other individual's who aided in reviewer Mary Ann TuliDo you understand and agree to our policy of having open and named reviews, and having your review included with the published papers. (If no, please inform the editor that you cannot review this manuscript.)YesIs the language of sufficient quality?YesPlease add additional comments on language quality to clarify if needed
NAAre all data available and do they match the descriptions in the paper? YesAdditional CommentsAs with the other manuscripts, OGS v3 is mentioned, but this is not get available from the CGEN.
The data underlying Fig 4 and Fig5 are available.Are the data and metadata consistent with relevant minimum information or reporting standards? See GigaDB checklists for examples &lt;a href="http://gigadb.org/site/guide" target="_blank"&gt;http://gigadb.org/site/guide&lt;/a&gt;YesAdditional CommentsIs the data acquisition clear, complete and methodologically sound?YesAdditional CommentsIs there sufficient detail in the methods and data-processing steps to allow reproduction?YesAdditional CommentsIs there sufficient data validation and statistical analyses of data quality? YesAdditional CommentsIs the validation suitable for this type of data?YesAdditional CommentsIs there sufficient information for others to reuse this dataset or integrate it with other data?YesAdditional CommentsAny Additional Overall Comments to the AuthorThis manuscript is a comprehensive description of the manual curation of the ubiquitin proteasome pathway gene, with clear aims and methodology. RecommendationAccept


# Reviewer report: Review MS


Reviewer name and names of any other individual's who aided in reviewer Subhas HajeriDo you understand and agree to our policy of having open and named reviews, and having your review included with the published papers. (If no, please inform the editor that you cannot review this manuscript.)YesIs the language of sufficient quality?YesPlease add additional comments on language quality to clarify if needed
Are all data available and do they match the descriptions in the paper? YesAdditional CommentsAre the data and metadata consistent with relevant minimum information or reporting standards? See GigaDB checklists for examples &lt;a href="http://gigadb.org/site/guide" target="_blank"&gt;http://gigadb.org/site/guide&lt;/a&gt;YesAdditional CommentsIs the data acquisition clear, complete and methodologically sound?YesAdditional CommentsIs there sufficient detail in the methods and data-processing steps to allow reproduction?YesAdditional CommentsIs there sufficient data validation and statistical analyses of data quality? YesAdditional CommentsIs the validation suitable for this type of data?YesAdditional CommentsIs there sufficient information for others to reuse this dataset or integrate it with other data?YesAdditional CommentsAny Additional Overall Comments to the AuthorRecommendationAccept


# Editor report: Accept


Comments to the AuthorThank you for another paper in this series.
